# Rhizobacterial Strain *Bacillus* *megaterium* BOFC15 Induces Cellular Polyamine Changes that Improve Plant Growth and Drought Resistance

**DOI:** 10.3390/ijms17060976

**Published:** 2016-06-21

**Authors:** Cheng Zhou, Zhongyou Ma, Lin Zhu, Xin Xiao, Yue Xie, Jian Zhu, Jianfei Wang

**Affiliations:** 1 School of Life Science and Technology, Tongji University, Shanghai 200092, China; czhou1224@hotmail.com (C.Z.); putaojiuvsduyao@126.com (L.Z.); 2 Key Laboratory of Bio-Organic Fertilizer Creation, Ministry of Agriculture, Anhui Science and Technology University, Bengbu 233100, China; mazy@ahstu.edu.cn (Z.M.); xiaox@ahstu.edu.cn (X.X.); xiey@ahstu.edu.cn (Y.X.)

**Keywords:** *Bacillus megaterium*, polyamines, drought tolerance, abscisic acid, *Arabidopsis thaliana*

## Abstract

Plant-growth-promoting rhizobacteria can improve plant growth, development, and stress adaptation. However, the underlying mechanisms are still largely unclear. We investigated the effects of *Bacillus* *megaterium* BOFC15 on *Arabidopsis* plants. BOFC15 produced and secreted spermidine (Spd), a type of polyamine (PA) that plays an important role in plant growth. Moreover, BOFC15 induced changes in the cellular PAs of plants that promoted an increase of free Spd and spermine levels. However, these effects were remarkably abolished by the addition of dicyclohexylamine (DCHA), a Spd biosynthetic inhibitor. Additionally, the inoculation with BOFC15 remarkably increased plant biomass, improved root system architecture, and augmented photosynthetic capacity. Inoculated plants also displayed stronger ability to tolerate drought stress than non-inoculated (control) plants. Abscisic acid (ABA) content was notably higher in the inoculated plants than in the control plants under drought stress and polyethylene glycol (PEG)-induced stress conditions. However, the BOFC15-induced ABA synthesis was markedly inhibited by DCHA. Thus, microbial Spd participated in the modulation of the ABA levels. The Spd-producing BOFC15 improved plant drought tolerance, which was associated with altered cellular ABA levels and activated adaptive responses.

## 1. Introduction

In natural environments, plants are sessile organisms that are often under various abiotic stresses, such as drought, salinity, and extreme temperature. Unlike animals, plants cannot change their growth conditions to avoid detrimental effects of environmental stresses. Drought is one of the major limiting factors that affects plant growth, biomass, and production [1]. Climate model predictions indicate that global warming will gradually increase temperature, thereby escalating drought stress, in the near future [2]. Over the last decade, soil microbial communities colonized by plant rhizospheres have been gained considerable attention owing to the beneficial effects of rhizobacteria on plant growth and stress resistance [3,4]. Hence, rhizobacteria can greatly improve plant drought tolerance.

In plants, a series of complicated mechanisms have been evolved to cope with drought stress at morphological, physiological, and molecular levels. Abscisic acid (ABA) is an extensively studied hormone that plays cardinal roles in stress responses of plants [5]. Drought stress promotes ABA synthesis, which further induces stomatal closure and up-regulates the expression of ABA-responsive genes, thereby contributing to plant drought resistance [6]. Moreover, several important metabolites including osmolytes (such as proline and soluble sugar) and active molecules (such as polyamines and melatonin) accumulate substantially in drought-treated plants [7,8,9]. These molecules are involved in stabilizing membrane structures and regulating stress-responsive pathways. Among these metabolites, polyamines (PAs), such as spermidine (Spd), spermine (Spm), and putrescine (Put), ubiquitously exist in different plant species [10]. PAs have been found to play important roles in plant growth, development, and abiotic stress responses [11,12]. Moreover, increased cellular PAs significantly enhance the tolerance of plants to various abiotic stresses, such as drought, cold, or oxidative stress via the up-regulation of antioxidant enzymatic activities and stress-related genes [13,14,15].

In addition to the transgenic regulation of PAs synthesis, exogenous PAs also improve abiotic stress tolerance in plants. Spraying of Put in wheat improves drought resistance with increased photosynthetic traits, promotes osmolyte accumulation, and reduces membrane damage compared with the control plants [16]. Additionally, exogenous Spd induces the tolerance of *Panax ginseng* seedlings to high salinity by activating antioxidant enzymatic activities [17]. Interestingly, cellular PA levels have been found to influence the biosynthetic pathways of ABA. The application of Spd, Spm, and Put remarkably elevated the ABA content in the wheat grains under drought stress [18]. Exogenous Put promoted the ABA synthesis and enhanced drought tolerance of *Lotus tenuis* plants [19]. These studies have shown that PAs can adjust plant stress responses via the ABA-mediated signaling pathways. In view of the physiological functions of PAs, the biosynthesis of PAs has been genetically manipulated to enhance plant drought tolerance. However, transgenic plants must be subjected to rigorous food and environmental safety trials [20]. Moreover, almost all experiments on exogenous PA application were confined within the laboratory and small field regions because of the cost of PAs [16,17,18,19]. Thus, developing efficient ways to improve plant adaptation to drought stress has become an urgent concern.

The use of plant growth promoting rhizobacteria (PGPR) to induce plant drought resistance offers the following advantages [21]. (i) The cost of isolation, propagation and application of PGPR is low; (ii) some PGPR benefit diverse plant hosts, including monocots and dicot plants; in addition, (iii) transferring the ability of one stress-resistant plant species to another can easily manipulate through microbial inoculation. It is well documented that several species of rhizobacteria, such as *Bacillus* and *Paenibacillus*, are of paramount importance as PGPRs [22,23]. Some strains of *B. megaterium* (*Bacillus megaterium*) can improve plant growth and control pathogen invasion [24], but no information about the effects of *B. megaterium* on plant stress tolerance has been reported.

This study aimed to explore the effects of *B. megaterium* BOFC15 on plant growth and physiological responses under drought stress. Our results revealed that BOFC15 could release a mass of Spd into the growth medium. The BOFC15-inoculated plants displayed improved drought resistance with increased PA levels compared with those of the non-inoculated plants. This study not only investigated the mechanisms behind the regulation of plant drought stress responses by BOFC15, but also provides evidences that the Spd-producing microbes can increase the drought resistance of host plants.

## 2. Results

### 2.1. Analyses of BOFC15-Producing Polyamines (PAs) and Cellular PAs in Arabidopsis

**Figure 1:**
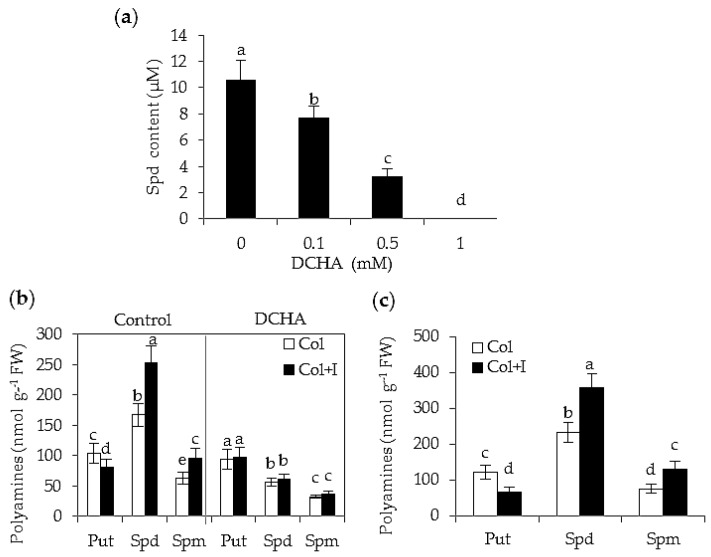
Determination of polyamines in the culture filtrates of BOFC15 and *Arabidopsis* plants. (**a**) Analysis of bacterial spermidine (Spd) under different concentrations of dicyclohexylamine (DCHA) (0, 0.1, 0.5 or 1 mM); (**b**) quantification of free polyamines (PAs) content of host plants grown in Murashige and Skoog (MS) agar medium with or without 1 mM DCHA after 5 d of BOFC15 treatments; and (**c**) quantification of polyamines (PAs) content of host plants grown in soils after 10 d of BOFC15 treatments. Col, control plants; I, inoculation with BOFC15; Col + I, inoculated plants; FW, fresh weight; Spd, spermidine; Spm, spermine; Put, putrescine; different lowercase letters above the bars represent significant difference at *p* < 0.05.

Put and Spd are two main types of PAs that can be synthesized by some bacteria [25]. To examine the types of PAs in the culture filtrates of BOFC15, high performance liquid chromatography (HPLC) analyses were carried out. Put was not detected, whereas Spd was detected in the culture filtrates of BOFC15 with a corresponding concentration was about 10.6 μM. In addition, the biosynthesis of bacterial Spd was analyzed in the presence of dicyclohexylamine (DCHA), an inhibitor of Spd synthase. The content of Spd in the culture filtrates of BOFC15 was gradually reduced along with increasing concentrations of DCHA. The biosynthesis of bacterial Spd was fully suppressed especially at 1 mM DCHA (Figure 1a).

To assess whether the inoculation with BOFC15 affected cellular PA levels in *Arabidopsis* plants, assays of plant-microbe interactions were performed, in which the *Arabidopsis* seedlings were cultured on Murashige and Skoog (MS) agar medium for 12 days (d), and the root vicinity of these plants were inoculated with BOFC15. The results of HPLC analyses indicated that the Spd and Spm content was increasingly elevated in the inoculated plants after 5 d of BOFC15 treatments, but the content of Put was distinctly reduced (Figure 1b). However, application of 1 mM DCHA abolished the BOFC15-induced increase of polyamines (Spd and Spm) in host plants (Figure 1b). In addition, the inoculation with BOFC15 induced marked changes in the PA levels of host plants after 10 d of BOFC15 treatments (Figure 1c).

### 2.2. BOFC15 Improves Plant Growth and Drought Tolerance

**Figure 2:**
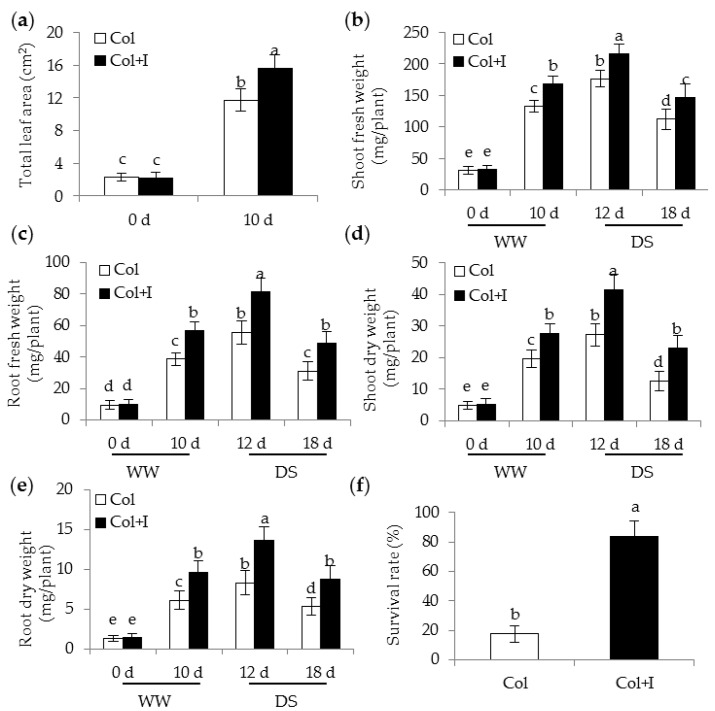
Inoculation with BOFC15 improved plant growth under well-watered (WW) and drought stress (DS) conditions. These plants were subjected to drought treatments for 12 and 18 d, respectively. Then, these plants were selected to analyze (**a**) total leaf area; (**b**) shoot fresh weight; (**c**) root fresh weight; (**d**) shoot dry weight; (**e**) root dry weight; and (**f**) survival rate. Col, control plants. Col + I, inoculated plants; different lowercase letters above the bars represent significant difference at *p* < 0.05.

To evaluate whether BOFC15 affected the growth and drought resistance of the *Arabidopsis* plants, the roots of each 12-day-old plant grown in plots with soils were initially inoculated with BOFC15. After 10 d of co-cultivation, several growth parameters, including leaf area (LA) and plant biomass, were determined in both the non-inoculated (control) and inoculated plants. Soil inoculation considerably increased the total LA of plants (Figure 2a). Moreover, the inoculation with BOFC15 resulted in 25.3% and 32.7% increase of above- and below-ground fresh weights (FWs), respectively (Figure 2b,c). Compared with the control plants, similar results were observed for the above- and below-ground dry weights (DWs) of inoculated plants, which were increased by 27.8% and 37.2%, respectively (Figure 2d,e).

**Figure 3:**
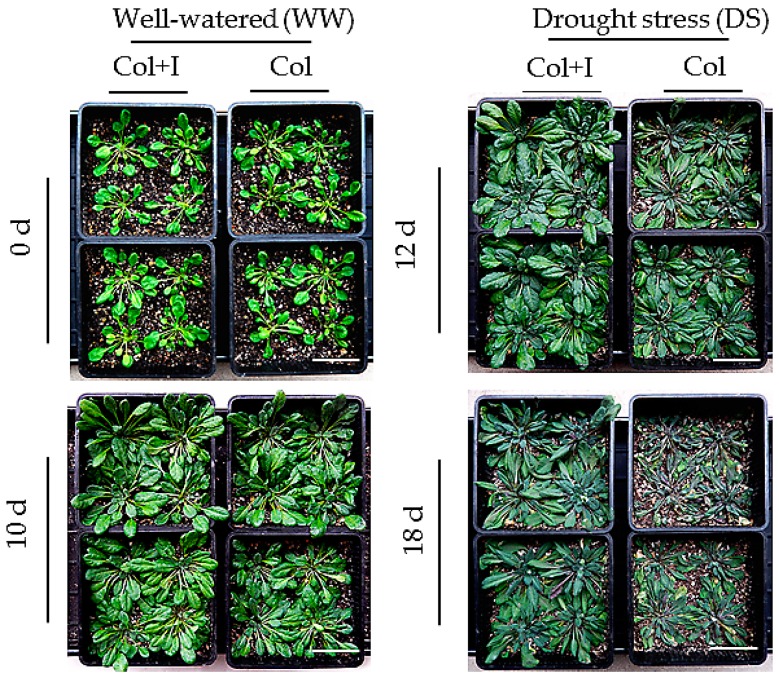
Inoculation with BOFC15 increased drought resistance in *Arabidopsis* plants. The control and inoculated plants were regularly watered for 10 d, and were then subjected drought treatment for 12 and 18 d, respectively; scale bars = 2 cm.

Furthermore, these plants suffered from drought treatments imposed by withholding water irrigation (Figure 3). After 12 d of water deprivation, the control plants produced curly and wilting leaves, whereas most of the inoculated leaves remained full and green. After 18 d without watering, the control plants exhibited more severe symptoms of wilting leaves than the inoculated plants. In addition, soil inoculation caused a relatively higher increase in plant size and biomass. The above- and below-ground FWs or DWs of inoculated plants were remarkably higher than those of the control plants. After 18 d of drought treatments, these plants were re-watered for 10 d to assess their survival rate. Our data showed that the inoculated plants had a distinctly higher survival rate (83.7%) than that of the control plants (18.6%) (Figure 2f). These results suggested that BOFC15 promoted plant growth and enhanced plant adaptation to water deprivation.

### 2.3. BOFC15 Incudes Alteration in Root System Architecture

**Figure 4:**
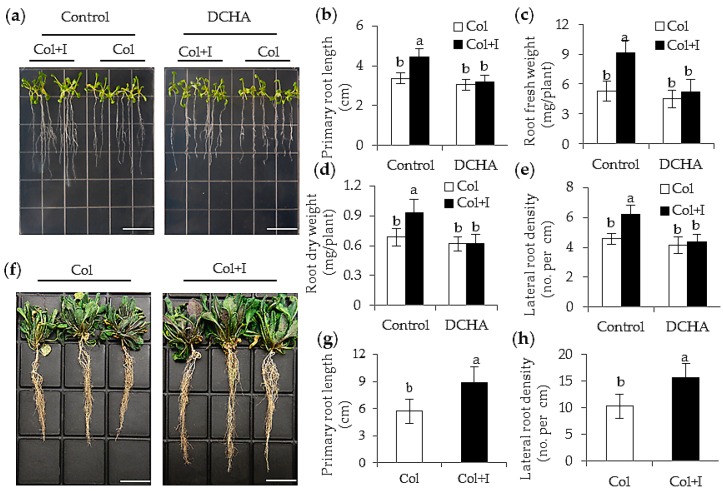
Inoculation with BOFC15 promoted plant root growth. 12-day-old *Arabidopsis* plants grown on MS agar medium were inoculated with BOFC15, and were then co-cultured for 6 d. Control, MS medium; DCHA, MS medium with 1 mM DCHA. These plants were selected to analyze several physiological parameters. (**a**) Phenotypes of plants grown in MS agar medium with or without DCHA; (**b**) primary root length; (**c**) root fresh weight; (**d**) root dry weight; (**e**) lateral root density; (**f**) phenotypic traits; (**g**) primary root length; and (**h**) lateral root density were examined in 22-day-old control (Col) and inoculated plants (Col + I) grown in soils; different lowercase letters above the bars represent significant difference at *p* < 0.05. Scale bars = 2 cm.

To assess if the inoculation with BOFC15 affected the root architecture of host plants, several characteristics of root growth were examined in both the control and inoculated plants. After 6 d of bacterial inoculation, the primary roots of the inoculated plants were remarkable longer than those of the control plants grown on MS agar plates. The inoculated plants also displayed more lateral roots than the control plants (Figure 4a,b). For this reason, the inoculation with BOFC15 caused a marked increase in root biomass compared with the control plants (Figure 4c,d). Similarly, after 20 d of soil inoculation, the inoculated plants had longer primary roots and more lateral roots than the control plants (Figure 4f–h).

To further confirm whether the BOFC15-producing Spd affected root architecture, we monitored the effects of DCHA on the root growth of both the control and inoculated plants grown on MS agar plates. Treatment with 1 mM DCHA largely inhibited BOFC15-induced root primary elongation and lateral root formation (Figure 4b,e), indicating that Spd was involved in the regulation of the root growth of host plants. Thus, the improved root systems of the inoculated plants considerably enhanced the assimilation of water and nutrient elements, which might facilitate plants’ adaptation under water deficit conditions.

### 2.4. BOFC15 Affects Leaf Water Status under Drought Stress

**Figure 5:**
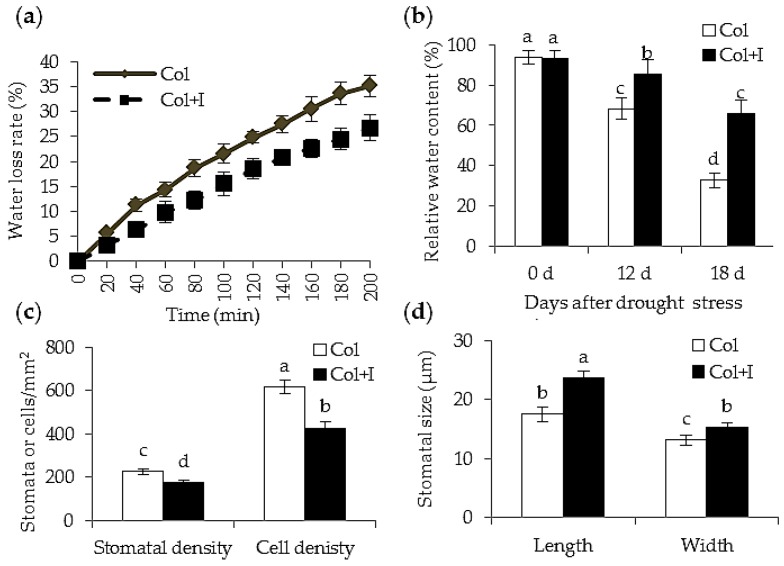
Inoculation with BOFC15 affected leaf water status under drought stress. 22-day-old *Arabidopsis* plants were subjected to water deprivation for 12 and 18 d, respectively. (**a**) Rosette leaves were detached from 22-day-old control (Col) and inoculated (Col + I) plants to monitor water loss rate; (**b**) rosette leaves from the control and inoculated plants under drought stress for 12 and 18 d were sampled to detect relative water content; In addition, the stomatal or cell density (**c**) and stomatal size (**d**) was measured in leaves of the control and inoculated plants; different lowercase letters above the bars represent significant difference at *p* < 0.05.

In this study, water loss rate of both the control and inoculated plants was examined. Rosette leaves were detached, and changes in their FWs were monitored over a 200 min period. The water loss rate was distinctly slower in the leaves of the inoculated plants than those in the control plants during the 200 min dehydration (Figure 5a). Relative water content (RWC) is a pivotal index for assessing plant water status, which is closely related to the adaptive ability of plants to water deficit [26]. Furthermore, the RWC in the leaves of both the control and inoculated plants was determined. In contrast to the inoculated plants, the RWC in leaves of the inoculated plants was increased by about 18.7% and 41.5% after 12 and 18 d of drought treatments, respectively (Figure 5b). To determine whether BOFC15 affected the stomatal features, the stomatal density and size were measured in leaves from the control and inoculated plants. The average stomatal density of the inoculated plants was about 18.2% less than that of the control plants (Figure 5c). However, the stomatal size was remarkably elevated in the inoculated plants (Figure 5d). The decreased stomatal density was likely caused by the expanded size of epidermal cells, showing a conspicuous reduction in cell density compared with the control plants. Thus, these data suggested that reduced water loss in the treated plants might be partially attributed to low stomatal density.

### 2.5. BOFC15 Increases Photosynthetic Capacity and Water Use Efficiency

**Figure 6:**
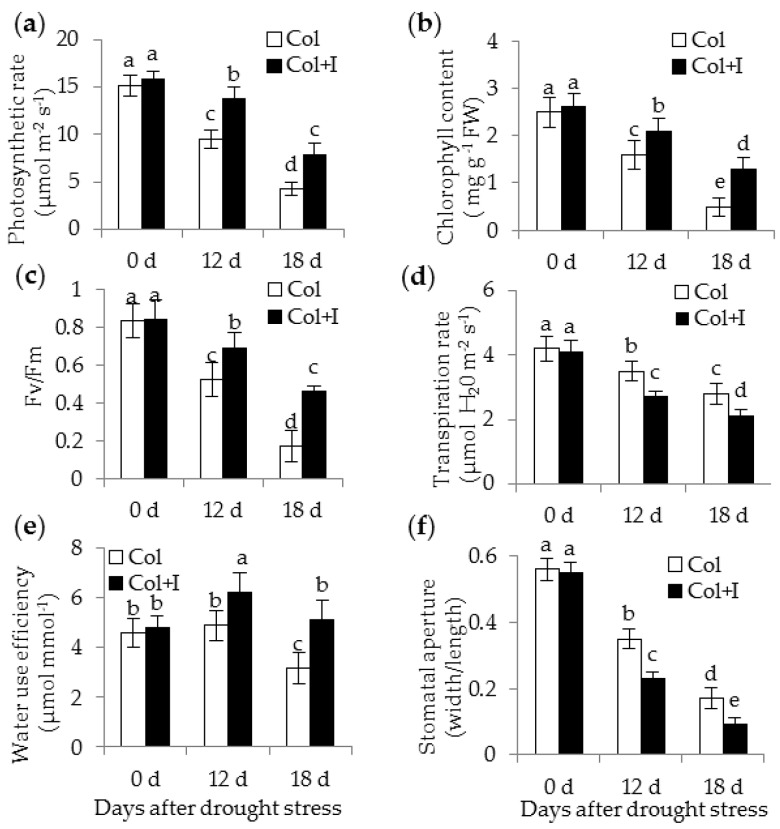
Inoculation with BOFC15 increased plant photosynthetic capacity and water use efficiency under drought stress. 22-day-old Arabidopsis plants were withheld from water for 12 and 18 d, respectively. Photosynthesis rate (**a**); chlorophyll content (**b**); maximum photosystem II quantum ratio of variable to maximum fluorescence (*F*v/*F*m) (**c**); transpiration rate (**d**); water use efficiency (**e**); and stomatal aperture (**f**) were analyzed in both the control (Col) and inoculated plants (Col + I); different lowercase letters above the bars represent significant difference at *p* < 0.05.

Stomatal density has been shown to influence photosynthetic traits and water use efficiency in plants [27]. In this study, several photosynthetic parameters were measured before and after drought treatments. The photosynthetic rates (Pn) of both the control and inoculated plants markedly dropped because of drought stress, but the value of Pn was considerably higher in the inoculated plants than in the control plants (Figure 6a). The chlorophyll content has been taken as a typical indicator that evaluates the ability of plants to tolerate abiotic stress [28]. Our data showed that the amount of chlorophyll displayed evident alterations with a gradual decrease accompanied by the time-delay of drought treatments (Figure 6b). The inoculated plants had higher chlorophyll content than the control plants after 12 or 18 d of water deprivation. Moreover, chlorophyll fluorescence was monitored in both the control and inoculated plants. The maximum photosystem II quantum ratio of variable to maximum fluorescence (*F*v/*F*m) in the control plants was notably lower than that in the inoculated plants (Figure 6c). Conversely, the control plants exhibited higher transpiration rates than the inoculated plants (Figure 6d), which contributed to higher water use efficiency in the inoculated plants (Figure 6e). Furthermore, the stomatal behavior was examined. Under well-watered (WW) conditions, there was no significant difference in the stomatal aperture between the control and inoculated plants. The stomatal aperture of the inoculated plants was 26.4% and 25.9% lower than those of the control plants after 12 and 18 d of drought treatments, respectively (Figure 6f).

### 2.6. BOFC15 Enhanced the Capability of Plants to Scavenge Oxygen Species (ROS)

**Figure 7:**
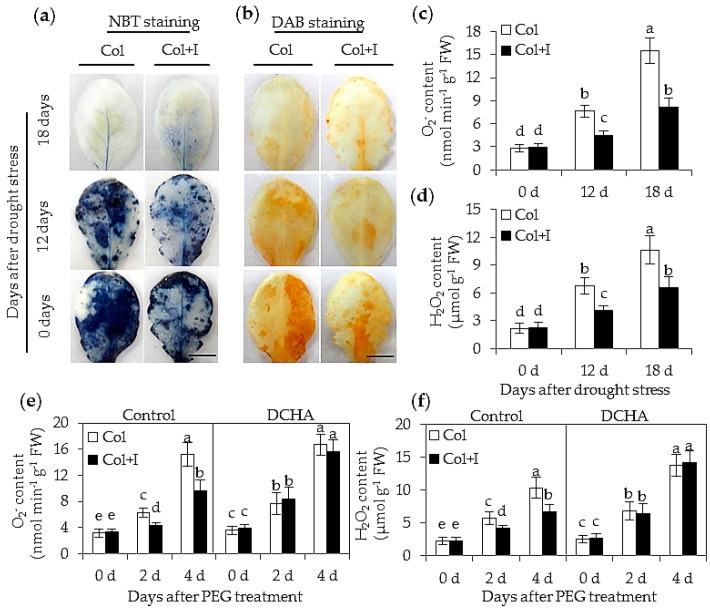
Inoculation with BOFC15 reduced cellular ROS levels in host plants under drought or PEG-treated stress conditions. 22-day-old plants experienced water deprivation for 12 and 18 d, respectively. Leaves were then separated to detected *in vivo* localization (**a**,**b**) and quantification (**c**,**d**) of O_2_^−^ and H_2_O_2_, respectively. Additionally, 12-day-old *Arabidopsis* seedling grown on MS medium with 10% PEG6000 was inoculated with BOFC15. After 2 or 4 d of PEG6000 treatments, the levels of O_2_^−^ (**e**) and H_2_O_2_ (**f**) were quantified in both the control (Col) and inoculated plants (Col + I). Control, MS medium; DCHA, MS medium containing 1 mM DCHA; different lowercase letters above the bars represent significant difference at *p* < 0.05. Scale bars = 1 cm (**a**,**b**).

The scavenging capacity for reactive oxygen species (ROS) is essential for plants to tolerate drought stress [29,30]. Two major types of ROS including O_2_^−^ and H_2_O_2_ were detected in both the control and inoculated plants. The leaves of the inoculated plants grown in soils exhibited more nitro blue tetrazolium (NBT) or 3,3′-diaminobenzidine (DAB) staining spots than those of the inoculated plants after 12 or 18 d without watering, but no significant difference was observed between the control and inoculated plants grown under WW conditions (Figure 7a,b). The control plants had more O_2_^−^ and H_2_O_2_ than the inoculated plants after 12 or 18 d of drought treatments (Figure 7c,d). To further ascertain whether the BOFC15-secreted Spd participated in cellular ROS detoxification, the ROS levels were quantified in both the control and inoculated plants grown on MS medium with 10% PEG6000 (Figure 7e,f). Lower ROS levels were observed in the inoculated plants than that of the control plants. However, the inoculated plants exhibited similar ROS level to the inoculated plants in the presence of 1 mM DCHA. These results indicated that microbial production of Spd played important roles in scavenging cellular ROS.

**Figure 8:**
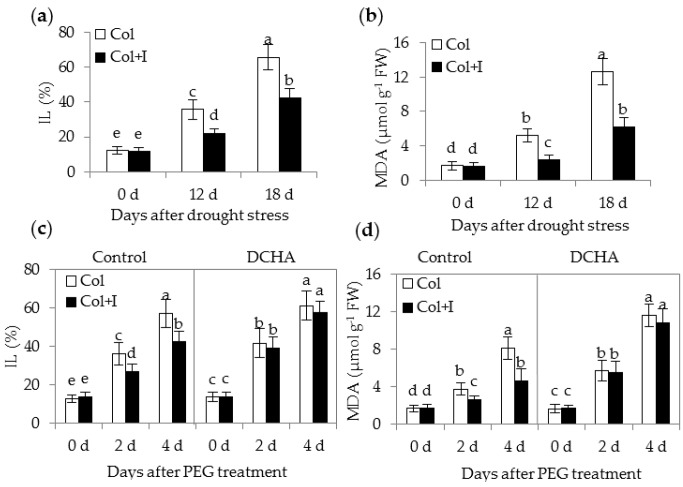
Inoculation with BOFC15 reduced the level of IL and MDA of host plants under drought or PEG-treated stress conditions. 22-day-old plants were subjected to water deprivation for 12 and 18 d, respectively. Leaves were then selected to measure values of IL (**a**) and MDA (**b**). Additionally, 12-day-old *Arabidopsis* seedling grown on MS medium with 10% PEG6000 was inoculated with BOFC15. After 2 or 4 d of PEG6000 treatments, the values of IL (**c**) and MDA (**d**) were determined in both the control (Col) and inoculated plants (Col + I). Control, MS medium; DCHA, MS medium containing 1 mM DCHA; different lowercase letters above the bars represent significant difference at *p* < 0.05.

Reduced ROS levels in the inoculated plants suggested that BOFC15 possibly mitigated oxidative injury to cellular structure under DS conditions. To assess the extent of the physical damage to membrane structure, the values of ion leakage (IL) and malonaldehyde (MDA) were determined in both the control and inoculated plants. The control plants had higher values of IL and MDA than the inoculated plants under DS conditions, whereas no apparent difference was observed between the control and inoculated plants grown under WW conditions (Figure 8a,b). Similarly, the inoculated plants displayed lower values of IL and MDA than the control plants when plants were grown under PEG-induced stress conditions, but no notable difference was observed between the control and inoculated plants in the presence of 1 mM DCHA (Figure 8c,d).

### 2.7. BOFC15 Increases Activities of Antioxidant Enzymes and Contents of Osmolytes

**Figure 9:**
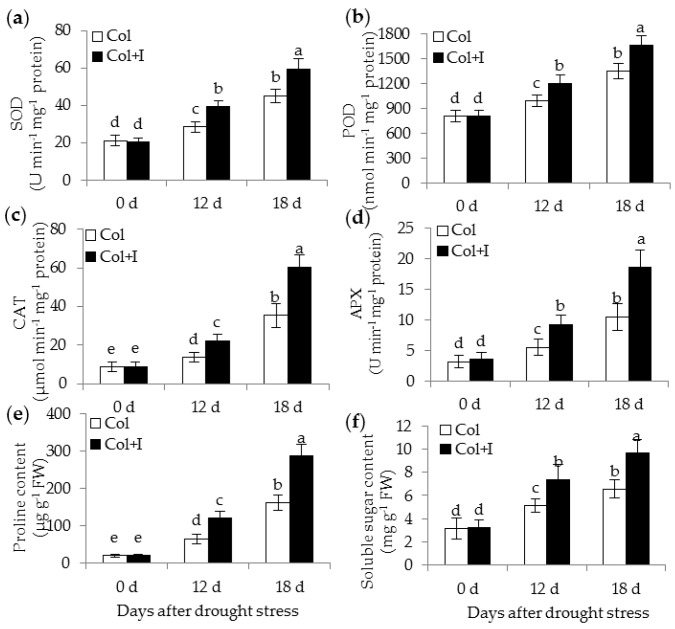
Inoculation with BOFC15 enhanced antioxidant enzymatic activities, and osmotic substances (proline and soluble sugar) in the *Arabidopsis* plants under drought stress. 22-day-old plants were subjected to water deprivation for 12 and 18 d, respectively. SOD (**a**); POD (**b**); CAT (**c**); APX (**d**); proline (**e**); and soluble sugar (**f**) were examined in both the control (Col) and inoculated plants (Col + I); different lowercase letters above the bars represent significant difference at *p* < 0.05.

Increased PA levels can improve drought tolerance by increasing antioxidant enzymatic activities [15]. Thus, the reduced ROS accumulation in the inoculated plants might be attributed to the activation of enzymatic antioxidant defense systems, implying that the BOFC15-secreted Spd possibly activated the enzymatic systems to eliminate ROS. As expected, several antioxidant enzymes including superoxide dismutase (SOD), peroxidase (POD), catalase (CAT), and ascorbate peroxidase (APX) showed remarkably higher activities in the inoculated plants than in the control plants after 12 or 18 d of drought treatments (Figure 9a–d).

Osmolytes, such as proline and soluble sugar, can mitigate drought-induced oxidative damages to cell structures [26]. Proline and soluble sugar content of the control plants was similar to those in the inoculated plants under WW conditions (Figure 9e,f). Furthermore, drought stress induced a more significant increase of proline and soluble sugar in the inoculated plants than in the control plants.

### 2.8. BOFC15 Up-Regulates the Expression of Abscisic Acid (ABA) Biosynthetic, Siginalling and Responsive Genes

**Figure 10:**
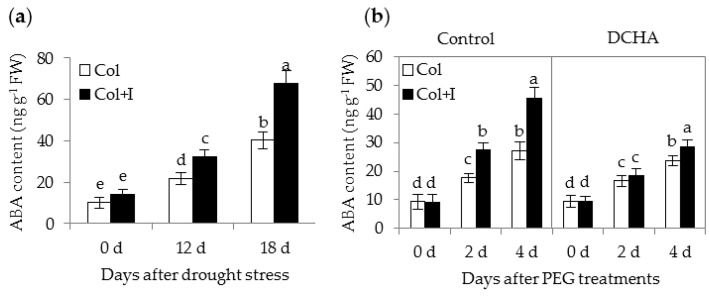
Inoculation with BOFC15 affected the ABA content in host plants under drought or PEG-treated stress conditions. (**a**) The ABA content was determined in leaves from both the control and inoculated plants grown under WW and DS for 12 or 18 d, respectively. Additionally, 12-day-old *Arabidopsis* seedling grown on MS medium containing 10% PEG6000 was inoculated with BOFC15; After 2 or 4 d of PEG6000 treatments, (**b**) the ABA content was quantified in both the control (Col) and inoculated plants (Col + I); different lowercase letters above the bars represent significant difference at *p* < 0.05.

ABA plays pivotal roles in regulating plant responses and tolerance to drought stress [5]. Importantly, increased PA levels affect the ABA content of drought-treated plants [18,19]. The inoculation with BOFC15 markedly altered cellular PA levels in *Arabidopsis* plants, indicating that the metabolism of ABA might be affected. Thus, the ABA content was quantified in both the control and inoculated plants by using the ELISA method (Figure 10a). The ABA levels in the inoculated leaves were slightly increased compared with those of the control plants under WW conditions. As a result of drought stress, the control plants displayed increased ABA content. Under DS conditions, the accumulation of ABA in inoculated plants was much higher than that in the control plants. The increase of ABA levels in drought-treated inoculated plants was consistent with the findings that soil inoculation remarkably reduced the water loss rate in the leaves of host plants.

Assays were conducted to investigate the relationship between BOFC15-secreted Spd and ABA synthesis. The control and inoculated plants were cultured on MS agar medium containing 10% PEG6000 with or without the presence of 1 mM DCHA (Figure 10b). The endogenous ABA content was distinctly elevated in both the control and inoculated plants grown under PEG-induced stress conditions, although it was obviously higher in the inoculated plants than in the control plants. Moreover, the BOFC15-induced ABA synthesis was largely inhibited in the presence of 1 mM DCHA, indicating that the BOFC15-secreted Spd directly or indirectly regulated the ABA synthesis of host plants.

**Figure 11:**
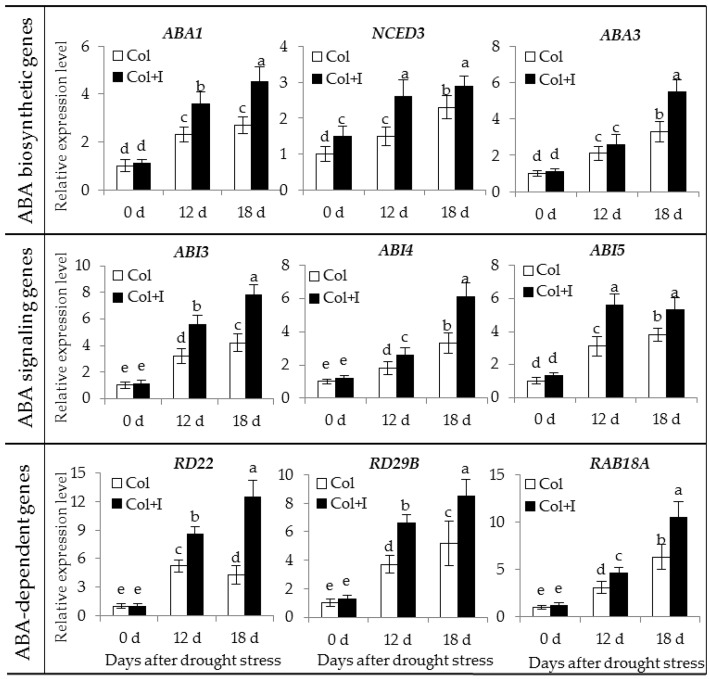
Inoculation with BOFC15 up-regulated the expression of ABA biosynthetic (*ABA1*, *NCED3*, and *ABA3*), signaling (*ABI3*, *ABI4*, and *ABI5*), or ABA-responsive genes (*RD22*, *RD29B*, and *RAB18A*) in the *Arabidopsis* plants under DS conditions. 22-day-old plants were subjected to water deprivation for 12 and 18 d, respectively. Then, the expression of these ABA-related genes was analyzed in both the control (Col) and inoculated plants (Col + I); different lowercase letters above the bars represent significant difference at *p* < 0.05.

The transcription levels of several marked genes including ABA biosynthetic (*ABA1*, *NCED3*, and *ABA3*), signaling (*ABI3*, *ABI4*, and *ABI5*), and responsive genes (*RD22, RD29B*, and *RAB18A*) were analyzed in both the control and inoculated plants. The expression levels of all tested genes were prominently higher in the inoculated plants than in the control plants under DS conditions. In addition, the inoculated plants accumulated more transcripts of *NCED3* than the control plants under WW conditions. However, no significant difference was observed between the other ABA-biosynthetic gene expression levels of the control and inoculated plants (Figure 11).

## 3. Discussion

Some strains of PGPR play crucial roles in assist plants to cope with unfavorable conditions, including drought [31,32,33]. Among these PGPR strains, *Pseudomonas chlororaphis* O6 induces systemic tolerance to drought stress in *Arabidopsis* plants through a salicylic acid-dependent signaling pathway [31]. *B. subitilis* GB03 improved plant drought tolerance by increased accumulation of osmoprotectant [32]. A recent study reported *Phyllobacterium brassicacearum* STM196 increased the resistance of *Arabidopsis* plants to severe water deprivation by altering leaf ABA levels [33]. However, the mechanisms behind the microbially enhanced plant stress tolerance remain unclear because of the different functions of diverse PGPR strains. Herein, we first reported a novel mechanism by which *B. megaterium* BOFC15 conferred increased plant drought resistance through the activation of PA-mediated signaling pathways. This mechanism involved the regulation of root system architecture and ABA-related stress responses.

### 3.1. Bacterial Spd Plays Important Roles in the Regulation of Plant Growth

The results of HPLC analyses indicated that BOFC15 could produce and secrete Spd, whereas the other types of Pas, including Spm and Put, were barely detected in the bacterial growth medium. Similarly, *B. subtilis* OKB105 can release abundant Spd into the growth medium, which promotes the root growth of tobacco plants [34]. In this study, the *Arabidopsis* plants inoculated with the Spd-producing BOFC15 had longer primary roots and more lateral roots than the control plants grown in soils. Consistently, robust root systems were also observed in the inoculated plants grown in the MS agar medium. However, the promoting effects of BOFC15 on plant root growth were severely suppressed by DCHA. Recently, the biosynthesis of Spd was shown to be significantly inhibited in the growth medium of *B. subtilis* OKB105 in the presence of DCHA [34]. In addition, treatment with DCHA abolished the BOFC15-induced increase of polyamines (Spd and Spm) in host plants. Why did the application of DCHA inhibit the observable effects caused by the inoculation with BOFC15? Possibly, microbial Spd was initially transported into plant cells, and some of them could be used as important precursors to produce Spm by spermine synthase [35], which eventually increased cellular Spd and Spm of host plants. Upon exposure to DCHA, the synthesis of microbial Spd and endogenous Spd in host plants was severely hampered. The application of exogenous PAs can remarkably increase the resistance of plants to various abiotic stresses, as shown in the literature [16,17,18,19]. Therefore, BOFC15-producing Spd induced a significant increase in cellular PAs, which contributed to the drought tolerance of host plants.

A deep and robust root system has been demonstrated to be conducive for the absorption of water from deep soils, which is an important indicator of drought tolerance in plants [31]. Thus, the BOFC15-secreted Spd could improve root system architecture to facilitate the drought tolerance of plants. In addition, the *Arabidopsis* plants displayed increased rosette LA after 10 d of BOFC15 treatments, which may result from the deep and robust root systems that enhanced the ability of plants to absorb nutrients and water. Our data are similar with the results shown by several recent studies. The PGPR strains, such as *Azospirillum lipoferum* USA 5 [36] and *A. brasilense* sp. 245 [37], promoted plant growth and increased biomass with augmented LA. *Burkholderia phytofirmans* PsJN-treated *Arabidopsis* plants also exhibited more rosette LA than the control plants [38]. Furthermore, it was also found that the RWC was similar in the control and inoculated plants under WW conditions, but it was much higher in the inoculated plants than the control plants under DS conditions. In addition, it is noteworthy that the inoculations with BOFC15 led to a significant increase of water use efficiency in host plants under DS conditions. Therefore, these morphological and physiological changes in host plants were benefited for retaining better water status under DS conditions.

### 3.2. BOFC15 Confers Plant Drought Tolerance, Which Correlates with Cellular PA Levels

Cellular PAs is one of the most important hallmarks of plant adaptation to environmental stresses [12,13,14,15]. In this study, the cellular Spd and Spm content were remarkably higher in the BOFC15-inoculated plants than in the control plants. We found that the inoculated plants had stronger tolerance to drought stress than the control plants. The chlorophyll content and photosynthetic efficiency are generally taken as the primary indexes of plant stress tolerance [29,30]. Inoculated plants had more chlorophyll content than the control plants under DS conditions, but no significant difference was observed between the control and inoculated plants under WW conditions. Furthermore, the chlorophyll fluorescence parameter *F*v/*F*m in both the control and inoculated plants gradually decreased following the duration of drought treatments. However, these photosynthetic values were dramatically higher in the inoculated plants than in the control plants, indicating that the former can maintain high photosynthetic capacity under DS conditions. Thus, the changing tendency of physiological parameters was associated with the ability of plants to cope with negative effects of drought stress. Our results were consistent with previous studies on high-level PAs that allowed the plants to withstand harsh conditions, including drought, salinity, oxidative stress, and heavy metal stress [12,13,14,15,16,17,18]. The data shown in this study imply that BOFC15 enhanced the ability of plants to tolerate drought stress, which was likely attributed to the altered cellular PA levels.

### 3.3. BOFC15 Confers Efficient Antioxidant Systems of Plants under Drought Stress

Drought stress frequently triggers the overproduction of ROS, which causes cell membrane damages through the oxidization of lipids and proteins [30]. Moreover, PAs can directly bind several macromolecules, including proteins and nucleic acids, and protects them against ROS-induced oxidative injuries. Our data showed that the values of IL and MDA, which are important indexes of membrane damage [29,30], were much lower in the inoculated plants than in the control plants under DS conditions. Similarly, the inoculated plants had lower ROS production and MDA and IL levels than the control plants under PEG-induced stress conditions (mimic drought stress). To further analyze whether the BOFC15-secreted Spd was required to enhance the tolerance of plants to oxidative stress imposed by drought treatments, DCHA was used to inhibit the synthesis of Spd. In contrast to the control plants, the inoculation with BOFC15 did not reduce ROS accumulation and IL and MDA values in the presence of DCHA.

ROS are continuously generated during plant growth and development, and a highly coordinated homeostasis concurrently exists between ROS production and detoxification. However, the dynamic homeostasis is often destroyed under long-term stress conditions; in turn, the overproduction of ROS leads to oxidative injuries to membrane protein and lipid, and finally induces cell death [26,29,30]. PAs play crucial roles in modulating cellular ROS homeostasis through the up-regulation of antioxidant enzymatic activities. Exogenous PAs have been shown to elevate antioxidant enzymatic activities, accompanied by a significant decrease in the accumulation of ROS under drought stress [16,17]. Additionally, the overexpression of some PA-related biosynthetic genes increased the PA level and concomitantly improved plant stress tolerance with high antioxidant enzymatic activities [15,39,40]. Similar results were shown in the current study, in which activities of antioxidant enzymes including SOD, POD, CAT, and APX were notably higher in the BOFC15-inoculated plants than in the control plants. A recent study has reported that PAs can form complexes with SOD, GPX, and CAT, for which these enzymes function more efficiently compared with their isolated enzymes [41]. Moreover, exogenous PAs have been shown to enhance the activities of antioxidant enzymes such as SOD and CAT under abiotic stress conditions [16,17,42]. Hence, BOFC15 mitigated drought-induced oxidative damage to plant cells via the activation of antioxidant systems, thereby regulating the ROS generation and redox status.

### 3.4. Involvement of Abscisic Acid (ABA)-Mediated Pathways in BOFC15-Induced Plant Drought Resistance

ABA is the most important stress hormone that plays vital roles in plant response and tolerance to adverse conditions [5,6]. In this study, a marked increase in ABA content was observed in the drought-treated plants, whereas the ABA level was distinctly higher in the inoculated plants than in the control plants under DS conditions. Furthermore, no significant difference was observed in the ABA levels between the control and inoculated plants under WW conditions. However, the expression of *NCED3* was much higher in the inoculated plants than in the control plants. Recent studies have shown that the overexpression of one ABA-biosynthetic gene in transgenic plants cannot significantly promote ABA synthesis under normal conditions, whereas the transgenic plants had more ABA content than the control plants under DS conditions [29,43,44,45]. Indeed, drought stress can rapidly induce the transcription of several ABA-biosynthetic genes in plants. The increased expression of *ABA3* and other ABA biosynthetic genes caused a coordinated increase in *de novo* ABA synthesis of transgenic lines that overexpress *ABA3* under DS conditions [43,44,45]. Previous studies have shown that rapid the induction of the ABA synthesis was conducive to the reduction of water loss, increasing the accumulation of osmolytes and activating stress-related gene expression [46,47]. As expected, the high ABA content of the inoculated plants under drought stress conditions coincided with their physiological alterations, including decreased stomatal aperture, reduced water loss, and increased proline and soluble sugar levels. Moreover, the expression of several marker genes including ABA biosynthetic (*ABA1*, *NCED3*, and *ABA3*), signaling (*ABI3*, *ABI4*, and *ABI5*), and responsive genes (*RD22*, *RD29B*, and *RAB18A*) were significantly up-regulated in the inoculated plants compared with the control plants. These genes play important roles in enhancing abiotic stress tolerance [48,49,50]. Thus, the increase of cellular ABA levels was one of main factors that improved the drought tolerance of the host plants.

Based on the data shown in this study, the inoculation with BOFC15 increased plant drought resistance, which contributed to the effects caused by microbe-induced increase in cellular PAs, including improved root systems, enhanced antioxidant enzymatic activities, and increased ABA content. These observations were consistent with the results reported by previous studies; in particular, high PA levels can directly promote root growth [34] and up-regulate antioxidant enzymatic activities under stress conditions [40,41,42]. Moreover, the inoculated plants exhibited higher ABA contents than the control plants under DS conditions. Cohen *et al*. [36] indicated that the *A. brasilense*-induced increase of ABA content improved the drought tolerance in maize. Such observation was quite similar to our results. However, whether the BOFC15-induced systemic drought tolerance resulted from PA-mediated ABA synthesis was still unknown. The ABA level was much higher in the inoculated plants than in the control plants under PEG-induced stress conditions. No significant differences were observed in the ABA level between the control and inoculated plants in the presence of DCHA, thereby implying that the BOFC15-producing Spd plays an essential role in the regulation of ABA synthesis. Consistently, the other effects caused by the interaction of BOFC15 with host plants such as promoted root growth and alleviated drought-induced oxidative injuries were almost blocked by DCHA treatment. Therefore, BOFC15 conferred improved plant growth and drought tolerance primarily through PA-mediated signaling pathways.

## 4. Materials and Methods

### 4.1. Plant Material and Growth Conditions

*A. thaliana* (*Arabidopsis thaliana*, Columbia ecotype) seeds were surface sterilized and placed on MS agar medium in a growth chamber (Percival, Perry, IA, USA) at 21 ± 2 °C with a light/dark cycle of 16 h/8 h. At 12 d after germination, the *Arabidopsis* seedlings were transferred into plastic pots containing soil. In addition, 12-day-old seedlings were transferred into vertical MS agar plates to monitor plant root growth.

### 4.2. Bacterial Culture and Inoculation, and Drought Treatments

*B. megaterium* strain BOFC15 was isolated from cultivated wheat plants and identified by 16S rDNA sequencing (Genebank No. KU851253). This bacterial strain was maintained on half-strength MS solid medium with 2% sucrose and 0.8% agar. For bacterial inoculation experiments, BOFC15 was streamed into liquid nutrient medium and cultured in an orbital shaker (160 rpm) at 37 °C for 16 h. The bacteria were then collected through centrifugation at 6000× *g* for 15 min at 4 °C. Then, the precipitates were washed and diluted to an OD_600_ nm absorbance of 0.1 with sterile phosphate-buffered solution. The roots of 12-day-old seedlings were inoculated with 5 μL of bacterial diluent and placed on vertical MS agar plates. Furthermore, 1 mL of bacterial diluent was injected into the vicinity of the roots of each plant grown in soils. For drought treatments, the control and inoculated plants were regularly watered for 10 d and were subjected to progressive drought by withholding water irrigation for up to 18 d.

### 4.3. Measurement of PAs and ABA

Bacterial Spd and Spm were quantified according to the method described by Xie *et al.* [34]. Free PA content in plants was detected as reported by Zhou *et al.* [15]. In addition, the ABA content was detected as described by Lu *et al.* [43]. The control and inoculated plants grown on MS agar plates supplemented with 10% PEG 6000 were used to quantify the ABA content by using the plant ABA ELISA kit (Cusabio, Carlsbad, CA, USA).

### 4.4. Determination of Physiological and Biochemical Parameters

Several physiological parameters, including stomatal density, photosynthesis rate, transpiration rate, and water use efficiency were detected according to the methods described by Yu *et al.* [51]. Additionally, the chlorophyll content and photosynthetic efficiency (*F*v/*F*m) were measured as reported by Zhang *et al.* [52].

*In vivo* localization of ROS (O_2_^−^ and H_2_O_2_) in leaves from the control and inoculated plants was detected according to the method reported by Sun *et al.* [53]. Moreover, the content of O_2_^−^ and H_2_O_2_ was quantified as recently by Liu and Pang [54]. The MDA content was spectrophotometrically detected based on a thiobarbituric acid (TBA)-based colorimetric method reported by Heath and Packer [55]. The value of IL was analyzed according to the method described by Jiang and Zhang [56]. The concentration of proline and soluble sugar was quantified as described by Yadav *et al.* [57].

### 4.5. Detection of Antioxidant Enzymatic Activities

Activities of antioxidant enzymes were determined using a spectrophotometer [58]. The leaf sample (1.0 g) was ground in liquid nitrogen, and homogenized in 5 mL of 50 mM phosphate buffer (pH 7.8) containing 1% (*w*/*v*) polyvinylpyrrolidone. The homogenate was centrifuged at 12,000× *g* for 15 min at 4 °C, and the supernatant was collected to analyze antioxidant enzymatic activity analysis. The antioxidant enzymatic activities were spectrophotometrically measured using the SOD, POD, CAT, and APX Activity Assay Kit (Jiancheng, Nanjing, China) according to the manufacturer’s instruction.

### 4.6. Quantative PCR (qPCR) Analysis

Total RNA was extracted from the control and inoculated plants using TRIzol Reagent Kit (Invitrogen, Carlsbad, CA, USA). Then, total RNA (500 ng each) from these samples was reversely transcribed into cDNA. Quantative PCR (qPCR) analysis was performed using the SYBR^®^ Green qPCR kit (Takara, Dalian, China) according to the method reported by Zhu *et al.* [59]. The *Arabidopsis Actin2* gene (*AtActin2*) was selected as the internal standard. The primers used in this study were listed in Table S1.

### 4.7. Statistical Analyses

Each experiment was performed three times. The mean values ± SE of at least three replicates are indicated, and different letters represent significant differences between the control and BOFC15-inoculated plants within control or treated groups by using Tukey’s test (*p* < 0.05).

## 5. Conclusions

**Figure 12:**
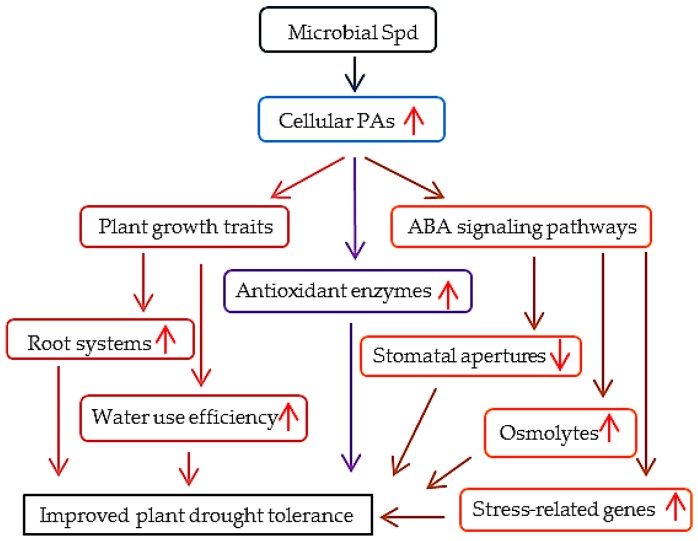
A proposed model illustrating the mechanism of BOFC15-induced plant drought resistance. The BOFC15-secreted Spd induces a remarkable increase in cellular PAs levels of host plants, thereby promoting plant growth and activating ABA-mediated signaling pathways under drought stress. These physiological, biochemical, and molecular alterations contribute to improving plant adaptation to drought stress. Red upright or downward arrows represent increase or decrease in concentrations or effect, respectively.

In sum, a model was proposed to illustrate the mechanisms of BOFC15-induced plant drought tolerance (Figure 12). The PGPR *B. megaterium* BOFC15 alleviated adverse effects of drought stress in *Arabidopsis* plants by increasing the accumulation of cellular PAs, which thereby activated a series of adaptive responses. Here, we verified the vital role of BOFC15 in enhancing plant drought tolerance and their potential utilization as a great strategy for sustainable agriculture. The involvement of microbial Spd in plant physiological responses against water deprivation, as well as their interaction with ABA signaling pathways, was also proven in this study. However, further research should be conducted to clarify the intricate crosstalk between PAs and ABA signaling pathways.

## Supplementary Materials

Supplementary materials can be found at http://www.mdpi.com/1422-0067/17/6/976/s1.

## Abbreviations

PGPR	Plant-growth-promoting rhizobacteria
DCHA	Dicyclohexylamine
ABA	Abscisic acid
Spd	Spermidine
Spm	Spermine
Put	Putrescine
PA	Polyamine
