# Supplementary material for: Rhizobacterial Strain Bacillus megaterium BOFC15 Induces Cellular Polyamine Changes that Improve Plant Growth and Drought Resistance
Source: Int J Mol Sci. 2016 Jun 21;17(6):976. doi: 10.3390/ijms17060976 (PMC4926508; doi:10.3390/ijms17060976)
Supplement: Supplementary file 1 [file ijms-17-00976-s001.pdf]

# Supplementary Materials: Rhizobacterial Strain *Bacillus megaterium* BOFC15 Induces Cellular Polyamines Changes that Improve Plant Growth and Drought Resistance

**Cheng Zhou, Zhongyou Ma, Lin Zhu, Xin Xiao, Yue Xie, Jian Zhu and Jianfei Wang**

**Table S1.** Primers used in this study.

| Gene | Usage | Sequence |
|---|---|---|
| *ABA1* | qPCR | 5′AGATTCTAGCACGTGCGGTT<br>5′AGCTTCACTACGGCCAAACA |
| *NCED3* | qPCR | 5′ATCTGCGCTTCACACTCCTC<br>5′TTAGCTCCGTTGCGCACATA |
| *ABA3* | qPCR | 5′TTTCCGTGGACCCAAGACAG<br>5′ATTGCACTCCGAGGGGAAAG |
| *ABI3* | qPCR | 5′TGTGACGACTCTTCTGGTGC<br>5′TCATCGGAACAACGACCTGG |
| *ABI4* | qPCR | 5′CAATCCGATTCCACCACCGA<br>5′AAGTACCAAGCCACTTGCGA |
| *ABI5* | qPCR | 5′TGAGACTGCGGCTAGACAAC<br>5′AGCAAACACCTGCCTGAACT |
| *RD22* | qPCR | 5′ACTTGGTAAATATCACGTCAGGG<br>5′CTGAGGTGTTCTTGTGGCATACC |
| *RD29B* | qPCR | 5′CCGACAAGAGGTGATGTGAA<br>5′GTGTAACCTAGCTTTGAGGCA |
| *RAB18A* | qPCR | 5′AGTATGACGAGTACGGAAATC<br>5′CCTTGTCCATCATCCGAGCTAG |
| *ACTIN2* | qPCR | 5′GAAATCACAGCACTTGCA<br>5′AGCCTTTGATCTTGAGAG |
